# Supplementary figures and images for: Dormitory of Physical and Engineering Sciences: Sleeping Beauties May Be Sleeping Innovations
Source: PLoS One. 2015 Oct 15;10(10):e0139786. doi: 10.1371/journal.pone.0139786 (PMC4607160; doi:10.1371/journal.pone.0139786)

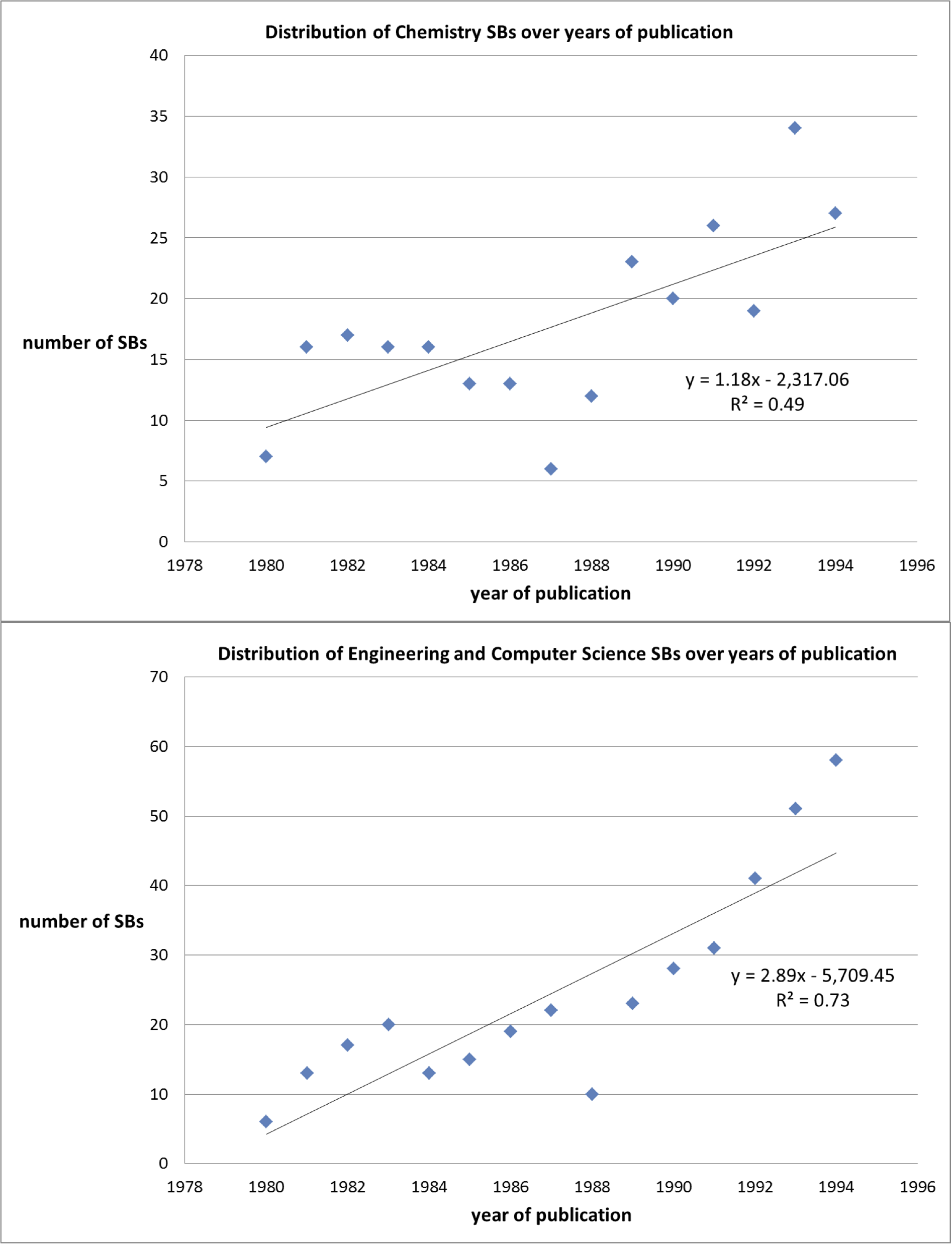

Supplement: S1 Fig — Upper part: chemistry; lower part: engineering & computer science. (TIF) [file pone.0139786.s001.tif]

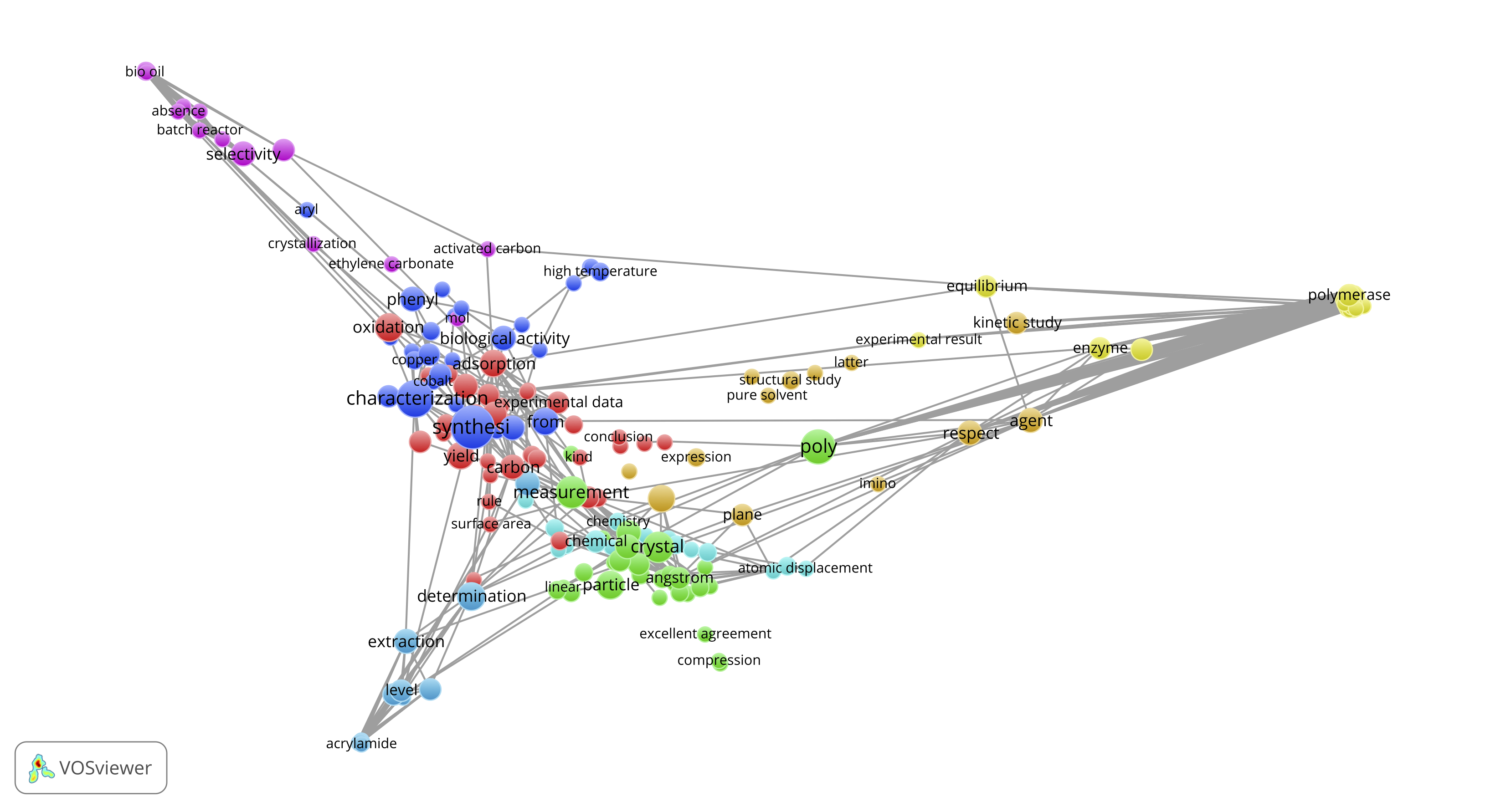

Supplement: S2 Fig — (TIF) [file pone.0139786.s002.tif]

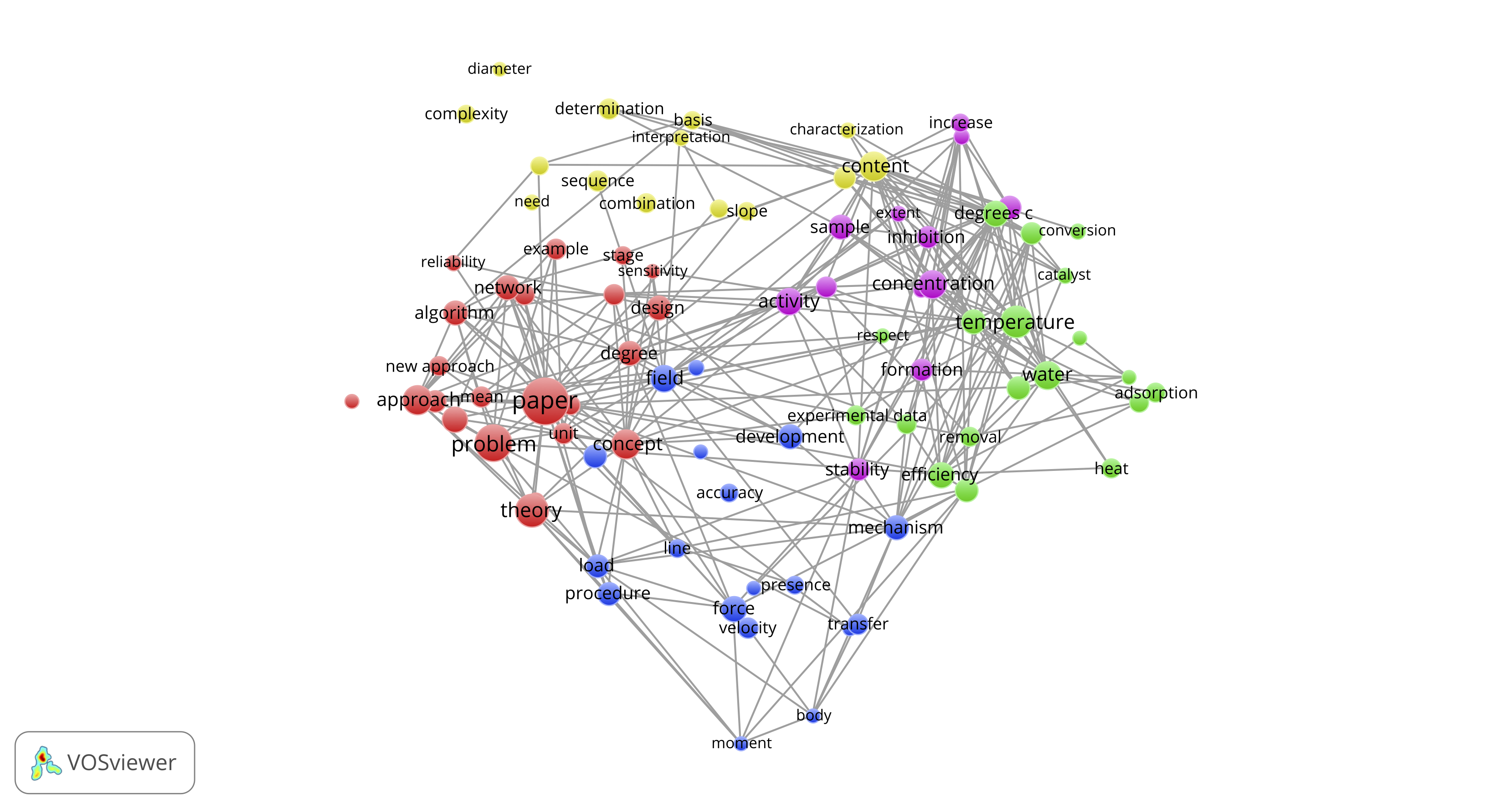

Supplement: S3 Fig — (TIF) [file pone.0139786.s003.tif]
