# Supplementary material for: Dormitory of Physical and Engineering Sciences: Sleeping Beauties May Be Sleeping Innovations
Source: PLoS One. 2015 Oct 15;10(10):e0139786. doi: 10.1371/journal.pone.0139786 (PMC4607160; doi:10.1371/journal.pone.0139786)
Supplement: S1 Table — (DOCX) [file pone.0139786.s005.docx]

**S1 Table**

*Results of the measuring procedure with 72 observations for chemistry*
