# Supplementary material for: Dormitory of Physical and Engineering Sciences: Sleeping Beauties May Be Sleeping Innovations
Source: PLoS One. 2015 Oct 15;10(10):e0139786. doi: 10.1371/journal.pone.0139786 (PMC4607160; doi:10.1371/journal.pone.0139786)
Supplement: S2 Table — (DOCX) [file pone.0139786.s006.docx]

**S2 Table**

*Definition of chemistry and engineering & computer science based on WoS journal categories (codes and field names)*

| **Chemistry** |  |
| --- | --- |
| 23 | BIOCHEMICAL RESEARCH METHODS |
| 24 | BIOCHEMISTRY & MOLECULAR BIOLOGY |
| 36 | CHEMISTRY, APPLIED |
| 37 | CHEMISTRY, MEDICINAL |
| 38 | CHEMISTRY, MULTIDISCIPLINARY |
| 39 | CHEMISTRY, ANALYTICAL |
| 40 | CHEMISTRY, INORGANIC & NUCLEAR |
| 41 | CHEMISTRY, ORGANIC |
| 42 | CHEMISTRY, PHYSICAL |
| 57 | CRYSTALLOGRAPHY |
| 63 | GEOCHEMISTRY & GEOPHYSICS |
| 71 | ELECTROCHEMISTRY |
| 198 | POLYMER SCIENCE |
|  |  |
|  |  |
| **Engineering & Computer Science** |  |
| 6 | ENGINEERING, AEROSPACE |
| 28 | BIOTECHNOLOGY & APPLIED MICROBIOLOGY |
| 44 | COMPUTER SCIENCE, ARTIFICIAL INTELLIGENCE |
| 46 | COMPUTER SCIENCE, CYBERNETICS |
| 47 | COMPUTER SCIENCE, HARDWARE & ARCHITECTURE |
| 48 | COMPUTER SCIENCE, INFORMATION SYSTEMS |
| 49 | COMMUNICATION |
| 50 | COMPUTER SCIENCE, INTERDISC APPLICATIONS |
| 51 | COMPUTER SCIENCE, SOFTWARE ENGINEERING |
| 52 | COMPUTER SCIENCE, THEORY & METHODS |
| 54 | CONSTRUCTION & BUILDING TECHNOLOGY |
| 75 | ENERGY & FUELS |
| 76 | ENGINEERING, MULTIDISCIPLINARY |
| 77 | ENGINEERING, BIOMEDICAL |
| 78 | ENGINEERING, ENVIRONMENTAL |
| 79 | ENGINEERING, CHEMICAL |
| 80 | ENGINEERING, INDUSTRIAL |
| 81 | ENGINEERING, MANUFACTURING |
| 82 | ENGINEERING, MARINE |
| 83 | ENGINEERING, CIVIL |
| 84 | ENGINEERING, OCEAN |
| 85 | ENGINEERING, PETROLEUM |
| 86 | ENGINEERING, ELECTRICAL & ELECTRONIC |
| 87 | ENGINEERING, MECHANICAL |
| 97 | FOOD SCIENCE & TECHNOLOGY |
| 119 | INSTRUMENTS & INSTRUMENTATION |
| 131 | OPERATIONS RESEARCH & MANAGEMENT SCIENCE |
| 145 | MEDICAL LABORATORY TECHNOLOGY |
| 147 | METALLURGY & METALLURGICAL ENGINEERING |
| 168 | NUCLEAR SCIENCE & TECHNOLOGY |
| 173 | REMOTE SENSING |
| 186 | IMAGING SCIENCE & PHOTOGRAPHIC TECHNOLOGY |
| 222 | TELECOMMUNICATIONS |
| 227 | TRANSPORTATION |
| 237 | MINING & MINERAL PROCESSING |
| 242 | TRANSPORTATION SCIENCE & TECHNOLOGY |
| 244 | AGRICULTURAL ENGINEERING |
| 245 | CRITICAL CARE MEDICINE |
| 247 | ENGINEERING, GEOLOGICAL |
| 248 | INTEGRATIVE & COMPLEMENTARY MEDICINE |
| 251 | ROBOTICS |
| 252 | NANOSCIENCE & NANOTECHNOLOGY |
| 257 | CELL & TISSUE ENGINEERING |
