# Supplementary material for: Dormitory of Physical and Engineering Sciences: Sleeping Beauties May Be Sleeping Innovations
Source: PLoS One. 2015 Oct 15;10(10):e0139786. doi: 10.1371/journal.pone.0139786 (PMC4607160; doi:10.1371/journal.pone.0139786)
Supplement: S3 Table — (DOCX) [file pone.0139786.s007.docx]

**S3 Table**

*Distribution of chemistry (upper table) and engineering & computer science SBs (lower table) over countries (first 15 countries are shown).*

***Chemistry***

| **Country** | **Number of SBs** | **% of total** |
| --- | --- | --- |
| USA | 85 | 32.1 |
| JAPAN | 35 | 13.2 |
| UK | 17 | 6.4 |
| FRANCE | 14 | 5.3 |
| FED REP GER | 13 | 4.9 |
| CANADA | 13 | 4.9 |
| SPAIN | 12 | 4.5 |
| PEOPLES R CHINA | 11 | 4.2 |
| AUSTRALIA | 9 | 3.4 |
| NETHERLANDS | 8 | 3.0 |
| ITALY | 8 | 3.0 |
| USSR | 5 | 1.9 |
| INDIA | 5 | 1.9 |
| SWEDEN | 4 | 1.5 |
| ISRAEL | 4 | 1.5 |

***Engineering & Computer Science***

| **Country** | **Number of SBs** | **% of total** |
| --- | --- | --- |
| USA | 139 | 37.9 |
| JAPAN | 43 | 11.7 |
| ENGLAND | 23 | 6.3 |
| CANADA | 21 | 5.7 |
| FRANCE | 20 | 5.5 |
| INDIA | 14 | 3.8 |
| NETHERLANDS | 11 | 3.0 |
| ISRAEL | 11 | 3.0 |
| ITALY | 10 | 2.7 |
| FED REP GER | 10 | 2.7 |
| AUSTRALIA | 8 | 2.2 |
| SPAIN | 7 | 1.9 |
| TAIWAN | 6 | 1.6 |
| SWEDEN | 6 | 1.6 |
| DENMARK | 4 | 1.1 |
