# Supplementary material for: Dormitory of Physical and Engineering Sciences: Sleeping Beauties May Be Sleeping Innovations
Source: PLoS One. 2015 Oct 15;10(10):e0139786. doi: 10.1371/journal.pone.0139786 (PMC4607160; doi:10.1371/journal.pone.0139786)
Supplement: S4 Table — Chemistry (upper table) and engineering & computer science SBs (lower table). (DOCX) [file pone.0139786.s008.docx]

**S4 Table**

*Institutions with three or more SBs. Chemistry (upper table) and engineering & computer science SBs (lower table).*

***Chemistry***

| **Institution** | **Number of SBs** | **% of total** |
| --- | --- | --- |
| UPJOHN CO | 5 | 1.9 |
| UNIV MUNICH | 5 | 1.9 |
| RUTGERS STATE UNIV | 5 | 1.9 |
| UNIV TEXAS | 4 | 1.5 |
| YAMANASHI UNIV | 3 | 1.1 |
| WASEDA UNIV | 3 | 1.1 |
| UNIV MELBOURNE | 3 | 1.1 |
| UNIV FLORIDA | 3 | 1.1 |
| UNIV CATHOLIQUE LOUVAIN | 3 | 1.1 |
| UNIV ALBERTA | 3 | 1.1 |
| UNIV ALABAMA | 3 | 1.1 |
| KINKI UNIV | 3 | 1.1 |
| CSIC | 3 | 1.1 |
| ACAD SCI USSR | 3 | 1.1 |

***Engineering & Computer Science***

| **Institution** | **Number of SBs** | **% of total** |
| --- | --- | --- |
| UNIV CALIF BERKELEY | 7 | 1.9 |
| IBM CORP | 7 | 1.9 |
| MIT | 6 | 1.6 |
| UNIV MASSACHUSETTS | 5 | 1.4 |
| TECHNION ISRAEL INST TECHNOL | 5 | 1.4 |
| UNIV TOKYO | 4 | 1.1 |
| UNIV CAMBRIDGE | 4 | 1.1 |
| CALTECH | 4 | 1.1 |
| USN | 3 | 0.8 |
| USAF | 3 | 0.8 |
| UNIV MINNESOTA | 3 | 0.8 |
| UNIV MARYLAND | 3 | 0.8 |
| UNIV FLORIDA | 3 | 0.8 |
| TOKYO INST TECHNOL | 3 | 0.8 |
| STANFORD UNIV | 3 | 0.8 |
| QUEENS UNIV BELFAST | 3 | 0.8 |
| OSAKA UNIV | 3 | 0.8 |
| NIPPON TELEGRAPH TEL PUBL CORP | 3 | 0.8 |
| LOUISIANA STATE UNIV | 3 | 0.8 |
| KYOTO UNIV | 3 | 0.8 |
| CSIC | 3 | 0.8 |
| BHARATHIAR UNIV | 3 | 0.8 |
