# Supplementary material for: Dormitory of Physical and Engineering Sciences: Sleeping Beauties May Be Sleeping Innovations
Source: PLoS One. 2015 Oct 15;10(10):e0139786. doi: 10.1371/journal.pone.0139786 (PMC4607160; doi:10.1371/journal.pone.0139786)
Supplement: S5 Table — Fields with ten or more SBs are shown. (DOCX) [file pone.0139786.s009.docx]

**S5 Table**

*Number of SBs in the chemistry fields (upper part) and engineering & computer science fields. Fields with ten or more SBs are shown.*

***Chemistry***

| **Field** | **Number of SBs** | **% of total** |
| --- | --- | --- |
| CHEMISTRY PHYSICAL | 60 | 22.6 |
| BIOCHEMISTRY MOLECULAR BIOLOGY | 46 | 17.4 |
| CHEMISTRY MULTIDISCIPLINARY | 45 | 17.0 |
| POLYMER SCIENCE | 32 | 12.1 |
| CHEMISTRY ORGANIC | 26 | 9.8 |
| CHEMISTRY APPLIED | 19 | 7.2 |
| CRYSTALLOGRAPHY | 18 | 6.8 |
| PHARMACOLOGY PHARMACY | 15 | 5.7 |
| FOOD SCIENCE TECHNOLOGY | 15 | 5.7 |
| CHEMISTRY MEDICINAL | 15 | 5.7 |
| GEOCHEMISTRY GEOPHYSICS | 13 | 4.9 |
| ELECTROCHEMISTRY | 13 | 4.9 |
| PHYSICS ATOMIC MOLECULAR CHEMICAL | 12 | 4.5 |
| CHEMISTRY ANALYTICAL | 11 | 4.2 |
| BIOPHYSICS | 11 | 4.2 |
| CHEMISTRY INORGANIC NUCLEAR | 10 | 3.8 |

***Engineering & Computer Science***

| **Field** | **Number of SBs** | **% of total** |
| --- | --- | --- |
| ENGINEERING ELECTRICAL ELECTRONIC | 74 | 20.2 |
| ENGINEERING CHEMICAL | 39 | 10.6 |
| FOOD SCIENCE TECHNOLOGY | 31 | 8.4 |
| ENGINEERING MECHANICAL | 28 | 7.6 |
| ENERGY FUELS | 25 | 6.8 |
| MATERIALS SCIENCE MULTIDISCIPLINARY | 23 | 6.3 |
| ENGINEERING CIVIL | 23 | 6.3 |
| ENGINEERING BIOMEDICAL | 23 | 6.3 |
| COMPUTER SCIENCE THEORY METHODS | 23 | 6.3 |
| OPERATIONS RESEARCH MANAGEMENT SC | 22 | 6.0 |
| MATHEMATICS APPLIED | 21 | 5.7 |
| BIOTECHNOLOGY APPLIED MICROBIOLOGY | 19 | 5.2 |
| PHYSICS APPLIED | 17 | 4.6 |
| METALLURGY METALLURGICAL ENGIN | 17 | 4.6 |
| CHEMISTRY APPLIED | 17 | 4.6 |
| TELECOMMUNICATIONS | 16 | 4.4 |
| MECHANICS | 16 | 4.4 |
| ENGINEERING MULTIDISCIPLINARY | 16 | 4.4 |
| COMPUTER SCIENCE SOFTWARE ENGIN | 13 | 3.5 |
| WATER RESOURCES | 12 | 3.3 |
| INSTRUMENTS INSTRUMENTATION | 12 | 3.3 |
| GEOSCIENCES MULTIDISCIPLINARY | 12 | 3.3 |
| TRANSPORTATION SCIENCE TECHNOLOGY | 11 | 3.0 |
| THERMODYNAMICS | 11 | 3.0 |
| STATISTICS PROBABILITY | 11 | 3.0 |
| COMPUTER SCIENCE INTERDISC APPLIC | 11 | 3.0 |
| MANAGEMENT | 10 | 2.7 |
| ENVIRONMENTAL SCIENCES | 10 | 2.7 |
| COMPUTER SC HARDWARE ARCHITECTURE | 10 | 2.7 |
