# Supplementary material for: Dormitory of Physical and Engineering Sciences: Sleeping Beauties May Be Sleeping Innovations
Source: PLoS One. 2015 Oct 15;10(10):e0139786. doi: 10.1371/journal.pone.0139786 (PMC4607160; doi:10.1371/journal.pone.0139786)
Supplement: S6 Table — (DOCX) [file pone.0139786.s010.docx]

**S6 Table**

*Distribution of the chemistry SBs (upper part) and engineering (lower part) over journals; journals with five or more SBs are shown.*

***Chemistry***

| **Journal** | **Number of SBs** | **% of total** |
| --- | --- | --- |
| JOURNAL OF BIOLOGICAL CHEMISTRY | 8 | 3.0 |
| JOURNAL OF APPLIED POLYMER SCIENCE | 8 | 3.0 |
| MACROMOLECULES | 7 | 2.6 |
| JOURNAL OF THE ELECTROCHEMICAL SOCIETY | 7 | 2.6 |
| TETRAHEDRON LETTERS | 6 | 2.3 |
| JOURNAL OF THE CHEMICAL SOCIETY FARADAY TRANSACTIONS I | 6 | 2.3 |
| JOURNAL OF THE AMERICAN OIL CHEMISTS SOCIETY | 6 | 2.3 |
| JOURNAL OF COLLOID AND INTERFACE SCIENCE | 6 | 2.3 |
| CHEMICAL PHYSICS LETTERS | 6 | 2.3 |
| PURE AND APPLIED CHEMISTRY | 5 | 1.9 |
| JOURNAL OF PHYSICAL CHEMISTRY | 5 | 1.9 |
| JOURNAL OF ORGANIC CHEMISTRY | 5 | 1.9 |
| JOURNAL OF AGRICULTURAL AND FOOD CHEMISTRY | 5 | 1.9 |

***Engineering & Computer Science***

| **Journal** | **Number of SBs** | **% of total** |
| --- | --- | --- |
| FUZZY SETS AND SYSTEMS | 11 | 3.0 |
| JOURNAL OF THE AMERICAN OIL CHEMISTS SOCIETY | 6 | 1.6 |
| IEEE TRANSACTIONS ON VEHICULAR TECHNOLOGY | 6 | 1.6 |
| JOURNAL OF AGRICULTURAL AND FOOD CHEMISTRY | 5 | 1.4 |
| IEEE TRANSACTIONS ON INFORMATION THEORY | 5 | 1.4 |
| ELECTRONICS LETTERS | 5 | 1.4 |
