# Supplementary material for: Dormitory of Physical and Engineering Sciences: Sleeping Beauties May Be Sleeping Innovations
Source: PLoS One. 2015 Oct 15;10(10):e0139786. doi: 10.1371/journal.pone.0139786 (PMC4607160; doi:10.1371/journal.pone.0139786)
Supplement: S7 Table — (DOCX) [file pone.0139786.s011.docx]

**S7 Table**

*Chemistry authors (upper table) and engineering & computer science authors (lower table) with at least three SBs.*

***Chemistry***

| **Authors** | **Number of SBs** | **% of total** |
| --- | --- | --- |
| CHOU KC | 5 | 1.9 |
| WARD WW | 4 | 1.5 |
| SUZUKI T | 3 | 1.1 |
| REUSSER F | 3 | 1.1 |
| LAURENT E | 3 | 1.1 |
| KIMURA Y | 3 | 1.1 |
| KEZDY FJ | 3 | 1.1 |
| DELMON B | 3 | 1.1 |

***Engineering & Computer Science***

| **Authors** | **Number of SBs** | **% of total** |
| --- | --- | --- |
| EPHREMIDES A | 3 | 0.8 |
| DEVOR RE | 3 | 0.8 |
| DAGANZO CF | 3 | 0.8 |
